# 18-FDG PET in lung transplantation

**DOI:** 10.1186/cc14337

**Published:** 2015-03-16

**Authors:** I Algieri, F Valenza, M Guanziroli, B Safaee Fakhr, M Cressoni, M Brioni, A Colombo, G Babini, F Crimella, D Massari, K Nikolla, G Crisafulli, S Paladini, L Rosso, A Palleschi, F Zito, D Chiumello, L Gattinoni

**Affiliations:** 1 Università degli Studi di Milano, Milan, Italy; 2 Fondazione IRCCS Ca' Granda-Ospedale Maggiore Policlinico, Milan, Italy

## Introduction

Lung transplantation is associated with an inflammatory reaction known as primary graft dysfunction. This clinical syndrome occurs within the first 72 hours after transplantation and is characterized by hypoxemia (PaO_2_/FiO_2_ <300) and bilateral infiltrates not secondary to cardiac dysfunction, viral or bacterial pneumonia and venous anastomotic obstruction.

## Methods

18-FDG PET scan was used to study 15 lung transplantation patients. The rate of 18-FDG uptake (Ki) was computed voxel by voxel with the Patlak method. Patients were divided according to the median Ki (27.8 (20.3 to 34.6) ml/minute/ml × 10^4^). Data are reported as median and interquartile range.

## Results

Five patients developed primary graft dysfunction; median Ki in these patients was not different from patients who did not (24.5(18.2 to 33.6) ml/minute/ml × 10^4^ vs. 29.1 (23 to 35.4) ml/minute/ ml × 10^4^ respectively, *P *= 0.64). Bilateral lung transplantation patients were characterized by a median Ki of 30.5 (22.9 to 34.5) ml/minute/ml × 10^4^, while patients undergoing single-lung transplantation presented a median Ki of 24.4 (21 to 34.1) ml/minute/ml × 10^4^ (*P *= 0.61). Considering single-lung transplantation, graft and native lung had similar Ki: 24.4 (21 to 34.1) ml/minute/ml × 10^4^ versus 24.2 (17.7 to 30.1) ml/minute/ml × 10^4^ respectively (*P *= 0.64). When patients were divided according to the median Ki value, higher Ki was associated with higher PaCO_2_ values (50 (46to 53) mmHg vs. 37 (34 to 44) mmHg, *P *= 0.01). See Table 1.

**Table 1:** 

	Ki <27.8 ml/minute/ml × 104 (*n *= 7)	Ki ≥27.8 ml/× minute/ml 104 (*n *= 8)	*P *value
PaO_2_/FiO_2_	280 (261 to 346)	239 (212 to 271)	0.31
pH	7.46 (7.43 to 7.48)	7.41 (7.39 to 7.44)	0.15
PaCO_2_ (mmHg)	37 (34 to 44)	50 (46 to 53)	0.01
WBCs (10^3^ cell/mm^3^)	8 (8 to 9)	9 (9 to 16)	0.34
Total lung volume (ml)	1,298 (1,092 to 1,494)	1,516 (1,408 to 1,665)	0.18
Total lung weight (g)	592 (488 to 741)	732 (597 to 774)	0.39
Total lung gas (ml)	706 (551 to 843)	804 (755 to 1,080)	0.15
Not-inflated lung tissue (%)	25 (12 to 30)	23 (17 to 30)	0.95
Poorly inflated lung tissue (%)	34 (42 to 42)	31 (29 to 34)	0.39
Well-inflated lung tissue (%)	36 (27 to 47)	47 (35 to 52)	0.53

## Conclusion

Patients clinically defined as having primary graft dysfunction did not have an increased rate of 18-FDG uptake. 18-FDG uptake was not different in single-lung versus bilateral transplantation and, in single-lung procedures, the native lung showed elevated inflammatory activity.

